# Extreme Thermal Sensitivity and Pain-Induced Sensitization in a Fibromyalgia Patient

**DOI:** 10.1155/2010/912513

**Published:** 2010-02-23

**Authors:** Fong Wong, Anthony Rodrigues, Siegfried Schmidt, Charles J. Vierck, Andre P. Mauderli

**Affiliations:** ^1^Department of Prosthodontics, College of Dentistry, University of Florida, D11-006, P.O. Box 100435, FL 32610, USA; ^2^Tufts Medical School, College of Medicine, MA 02111, USA; ^3^College of Medicine, University of Florida, Community Health and Family Medicine, FL 32611, USA; ^4^Departments of Neuroscience and Orthodontics, Colleges of Medicine and Dentistry, University of Florida, FL 32611, USA

## Abstract

During the course of a psychophysical study of fibromyalgia syndrome (FMS), one of the subjects with a long history of headache and facial pain displayed an extraordinarily severe thermal allodynia. Her stimulus-response function for ratings of cutaneous heat pain revealed a sensitivity clearly beyond that of normal controls and most FMS subjects. Specially designed psychophysical methods showed that heat sensitivity sometimes increased dramatically within a series of stimuli. Prior exposure to moderate heat pain served as a trigger for allodynic ratings of series of normally neutral thermal stimulation. These observations document a case of breakthrough pain sensitivity with implications for mechanisms of FMS pain.

## 1. Introduction

Fibromyalgia syndrome (FMS) is characterized by widespread hyperalgesia and allodynia for mechanical and thermal stimuli of deep and superficial tissues [1]. The magnitude, distribution, and frequency of the clinical pain and the responses to experimental stimuli vary widely [2, 3] suggesting to some: (1) that FMS represents the upper portion of the normal distribution of pain sensitivity [4], (2) that individuals with FMS are predisposed by their genetic makeup and psychological history to exhibit exaggerated pain effect (catastrophize) [4, 5] and therefore (3) that chronic FMS represents a psychological pain state. According to these suppositions, the widespread hypersensitivity characteristic of FMS would be associated with excessive activation of cortical levels involved in affective interpretation of pain [6, 7], without abnormal processing within pain pathways extending from the periphery to the initial stages of cortical somatosensory processing. 

The FMS patient presented here drew our attention because of the clinical severity of her disease and an abnormal sensitivity to cutaneous thermal stimulation. This prompted us to thoroughly evaluate her pain sensitivity with psychophysical methods that can reveal abnormal processing within primary pain pathways. This patient's high sensitivity to thermal stimulation reveals pathophysiogical pain processing. The studies were conducted with informed consent and approval by the IRB of the University of Florida.

## 2. Clinical Description

The patient initially presented with complaint of “hurting all over and being extremely fatigued”. She met the ACR criteria for FMS with typical symptoms, as well as physical findings [8]. These symptoms included severe headaches, nonrestorative sleep without other findings of a primary sleep disorder, sensations of weakness in her upper and lower extremities, profound fatigue, hypotensive/fainting episodes, Reynaud's phenomenon with cold and clammy feet and hands, and TMJ disorder. Her past history was significant for migraine headache disorder with aura since childhood. She denied any previous trauma except a right hand fracture in 1998 without residuals. There was no history of sexual/verbal/physical abuse, mental health problems, or drug abuse. She noted slow but steadily increasing, generalized pain since her adolescence and reported that her headaches were largely uncontrolled during her childhood. Better control of pain was achieved with a variety of medications in her later years; however, she noted extreme generalized pain in 2001 as she was trying to come off medications. She has been on medroxyprogesterone (Depo Provera) for birth control.

Generalized pain and associated symptoms continue, despite a variety of treatments. In general, they have slowly worsened, and she has experienced increasing problems staying functional. She has not developed significant mental health comorbidities. She underwent neuropsychological evaluation twice, and there was no diagnosis of somatization disorder, depression, or anxiety disorder. She was found to have a learning disability (reading disorder).

There have been no signs of connective tissue disease: that is, synovitis, joint effusions, or deformities. Her joints reveal some hypermobility, which has always been present. She has developed some osteoarthritis of the knees, lower back and shoulders. She has FMS tender points ranging from 13/18–18/18, irritation to the occipital nerves, and ongoing myofascial pain (MFP) trigger point areas. Of note, she has developed more sympathetic nervous system signs, with diffuse skin mottling and cold and clammy hands and feet. Her vital signs, including blood pressure, respiratory rate, and weight have all remained stable and normal. However, her heart rate is elevated (102–120) and felt to be secondary to her medications, particularly cyclobenzaprine (Flexeril). Her laboratory studies were and have remained normal, including CBC, chemistry profile, sedimentation rate, antinuclear antibody, rheumatoid factor, highly sensitive C-reactive profile, Vitamin B12, and thyroid studies. 

Treatments have been multidisciplinary, including pharmacological and nonpharmacological procedures. Her current regimen has kept her functioning with regular activities of daily living (ADLs). She is taking: topiramate (Topamax) 50 mg bid, cyclobenzaprine (Flexeril) 20 mg qhs, hydrocodone/acetaminophen 10/500 1–2 q6–8 hours, zolpidem (Ambien CR) 12.5 qhs, sumatriptan/naprosyn (Treximet)1 p.o. PRN, sumatriptan injectable (Imitrex) PRN, promethazine (Phenergan) 25 mg 1 q8 hours PRN nausea/vomiting, medroxyprogesterone (Depo Provera), and supplements, including magnesium 750 mg qd and CoenzymeQ10 150 mg bid. 

Over the years, she has tried other medications with varying results and side effects: NSAIDs with significant GI upset, occasional naprosyn, gabapentin (Neurontin) with numbness and fogginess, pregabalin (Lyrica) with severe mental fogginess, duloxetine (Cymbalta) with headaches and nausea, citalopram (Celexa) with weird feelings, glucosamine and chondroitin no help, seroquetiapine (Seroquel) with severe headaches and could not think, mirtazepine (Remeron) no help, zolpidem (Ambien) lost its effectiveness, zolmitriptan (Zomig) with dizziness, rizatriptan (Maxalt) lost effectiveness, various beta blockers, for example, metoprolol, atenolol lost its effectiveness, all Cox 2 inhibitors caused side effects and were ineffective, meperidine (Demerol) with nausea, fluoxetine (Prozac) no help, and paroxetine (Paxil) lost its effectiveness over time.

Nonpharmacological treatments included cognitive behavioral therapy and pain management counseling (helped with pain coping), physical therapy (helped with intermittent exacerbations of the MFP), massage therapy (which made her condition more painful), trigger point injections and occipital nerve blocks (helped when she had typical occipital neuralgia findings), and dietary adjustments becoming a lacto vegetarian.

**Distribution of FMS Pain**

**Figure 1:**
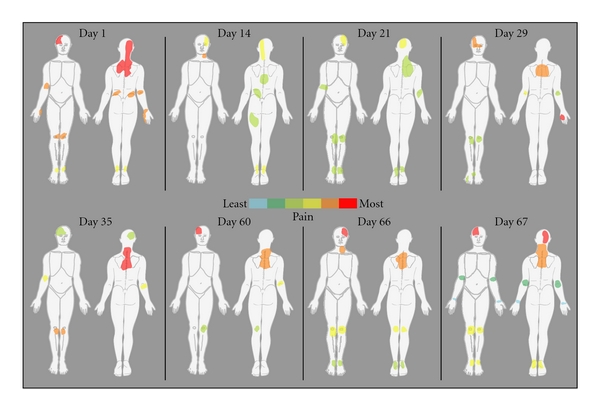
Spatial pattern of spontaneous pain of the FMS patient prior to psychophysical testing.

**Figure 2:**
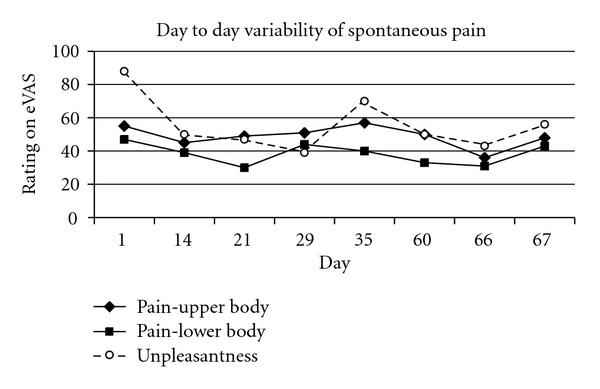
Spontaneous pain in the upper and lower body was rated with an electronic visual analog scale (eVAS) from 0 (no pain) to 100 (intolerably intense pain). The unpleasantness of the most severe pain overall was rated on a scale ranging from 0 (“not unpleasant”) to 100 (“the most unpleasant imaginable”).

At the beginning of each session (before experimental procedures were undertaken), the patient was asked to localize currently active pain sites on a diagram and rank the locations according to pain severity (Figures 1 and 2). In all cases pain was present in at least 3 quadrants of the body.

## 3. Study 1. Stimulus-Response Relationship (Single Stimuli)

### 3.1. Methods

In our initial attempt to evaluate the cutaneous pain sensitivity of the FMS patient, a solenoid-powered mechanism brought a Peltier-based thermode (23 × 23 mm) into and out of light skin contact. The hypothenar eminence of the left hand was contacted for 3 sec, with an inter-stimulus interval (ISI) of 30 sec. The temperature was set to 29°C at the beginning of the test. This is approximately 15°C below pain threshold of healthy individuals for this thermode size and stimulus duration. The temperature increased in 1°C increments for each contact until a pain rating of 50% was reached. The subject rated pain intensity by adjusting the slider of an electronic visual analog scale (eVAS) within 5 sec after the end of each pulse. The left and right endpoints of the slider were defined as “no pain” and “intolerably intense pain”, respectively. The position of the slider (and therefore pain intensity) was recorded as a numerical value from 0 to 100%.

### 3.2. Results

**Figure 3:**
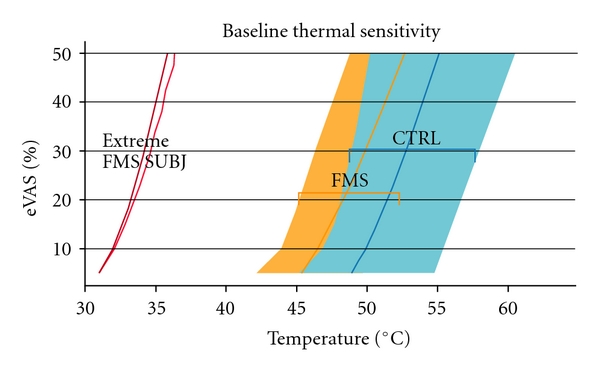
Series of thermal contact stimuli (pulse duration: 3 sec; ISI: 30 sec) were administered to the hypothenar eminence of the left hand. Red traces: S-R curves of the extremely sensitive FMS subject, acquired on two different days. For comparison, the mean curve-fitted (TableCurve2D by Systat, Chicago, IL), S-R curves from 12 typical FMS subjects (orange trace), and 12 healthy controls (blue trace) are presented. The borders of the colored bands represented the range of each group‘s data (from minimum to maximum). The thermal pain sensitivity of the severely sensitive FMS subject was far outside the range of the FMS group.

The stimulus-response (S-R) curve for the FMS patient (Figure 3) was far to the left of those of all other FMS patients we have tested. Thermode temperatures as low as 33.7°C were rated as moderately painful (25% on the eVAS) for the extremely sensitive FMS patient, compared to 49.2°C for FMS group and 52.2°C for the control group (group means). This standard psychophysical procedure demonstrated an extraordinary level of sensitization to thermal stimulation for the severe FMS patient of this study.

## 4. Study 2. Temporal Summation (Windup) of Thermal Pain

### 4.1. Methods

Repetitive series of thermal cutaneous stimuli can provide information on mechanisms for sensitized states of chronic pain patients. For example, temporal summation (windup) of thermal pain by stimuli that activate C nociceptors has been shown to result from NMDA-receptor-dependent central sensitization within pain pathways from the spinal cord to the primary somatosensory cortex [9–11]. Accordingly, the thenar eminence of the left hand (glabrous skin) of the FMS patient, was contacted repetitively with the solenoid driven thermode (23×23 mm) set to 41°C. Thermal pulse durations (PDs) of 0.7 sec were delivered at an interstimulus interval (ISI) of 2.5 sec.

### 4.2. Results

**Figure 4:**
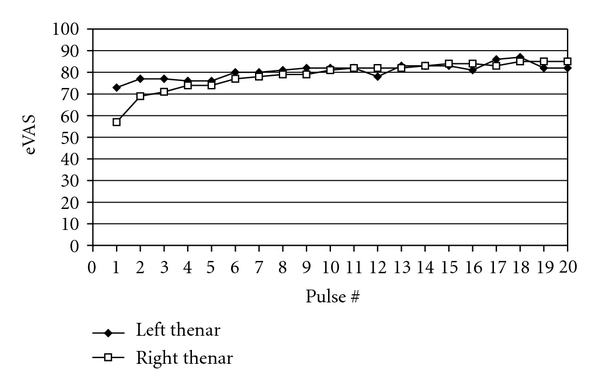
eVAS pain ratings of repetitive thermal stimulation (41°C, PD 0.7 sec, ISI 2.5 sec),on the right (open squares) and then the left (solid diamonds) thenar eminence of the FMS patient.

Normal subjects and typical FMS patients temporally summate when presented with a PD of 0.7 sec and an ISI of 2.5 second, but 49°C or higher temperatures are required. At these temperatures, ratings begin near zero and reach a plateau at moderate to high pain intensity levels after 10 to 15 stimuli [9, 12]. In contrast, ratings of pain intensity induced by the first stimulus to our FMS patient exceeded 50% and rapidly escalated to a plateau of approximately 85% (Figure 4).

## 5. Study 3. Rapid Transition to an Extreme Level of Sensitization

### 5.1. Methods

Ratings by the FMS patient of the first stimulus in the windup series revealed a high sensitivity to short pulses of heat stimulation that normally evoke pain only after repetition at a rate that progressively activates NMDA receptors (≤3 sec ISI) [9]. In order to further evaluate temporal summation by this sensitized patient, we utilized a new paradigm (designed by A.P.M.) that permits variation of a stimulus parameter within a series of stimuli. The fixed parameters were a PD of 0.7 sec and a temperature that elicited a low level of pain early in a series for the FMS patient (36°C). The remaining parameter, ISI, was adjusted by software as a function of the patient's eVAS rating. When windup occurred and pain intensity reached a predetermined set point (40%), ISI was increased from an initial value of 2.5 sec to maintain the ratings near the setpoint (pain intensity clamping). With this protocol, increasing ISIs indicate sensitization (prolonged temporal summation). 

### 5.2. Results

**Figure 5:**
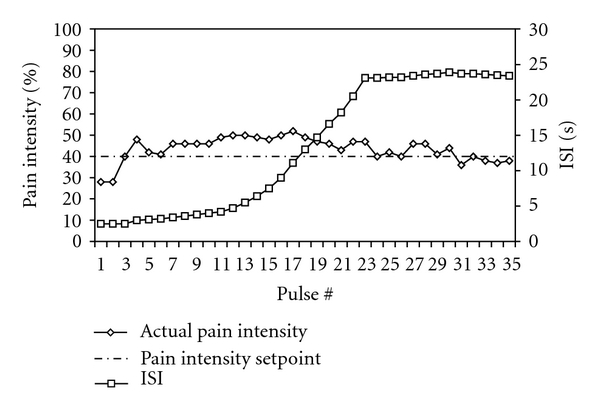
Pulse duration (0.7 sec) and thermode temperature (36°C) remained unchanged throughout a series of 35 stimuli. Pain intensity was clamped to 40% by adjusting ISI.

During the session shown in Figure 5 the thermode was at a temperature (36°C) that is barely perceived as warm and does not produce temporal summation in healthy individuals. For the FMS patient ISIs of 5 sec or less maintained pain levels near the 40% setpoint for the first 13 pulses of stimulation, and then substantial increases in ISI (to 24 sec) were required to maintain pain ratings near 40%. Thus, the FMS patient sensitized during stimulation at ISIs far beyond the longest that will maintain temporal summation of pain for a normal subject (approximately 6 sec) tested at a higher temperature [9]. Maintenance of temporal summation by infrequent stimulation has been demonstrated previously for FMS subjects [13], but it was extreme for the patient described here, considering the low temperature and long ISIs. 

## 6. Study 4. Sensitization Induced by Painful Stimulation

### 6.1. Methods

Pain intensity clamping indicated that the extraordinary thermal sensitivity of this patient increased further after receipt of painful stimulation. To determine whether pain is a determining factor for the allodynic states of this patient, three series of repetitive pulses (PD 0.7 sec, ISI 2.5 sec) were presented. The first series involved stimulation of the right thenar eminence with the thermode at skin temperature (32.5°C, measured with a Dermatemp infrared temperature scanner model DT-1001; Exergen Corp., Watertown, MA, USA). Ratings of sensation intensity remained near pain threshold (<10%) across the entire series. Second, a painful series of stimuli was presented for 8 minutes, with clamping of pain intensity at 40% by varying probe temperature.

### 6.2. Results

**Figure 6:**
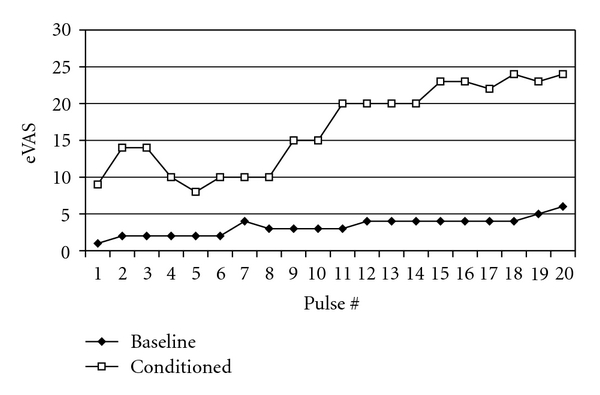
Identical pulse series (PD 0.7 sec, ISI 2.5 sec, thermode temperature same as measured skin temperature, i.e., 32.5°C) were administered to the right thenar eminence before (baseline) and after (conditioned) a series of painful stimulation. The intervening series of painful stimuli induced sensitization.

Following the painful second series, a third series of pulses was delivered at skin temperature. Pain intensity progressed from below 10% to approximately 25% (Figure 6). This suggests that the intervening (second) series of painful stimuli triggered (or enhanced) an allodynic state during the third series. 

## 7. Study 5. Thermal Stimulation without a Mechanical Component

### 7.1. Methods

**Figure 7:**
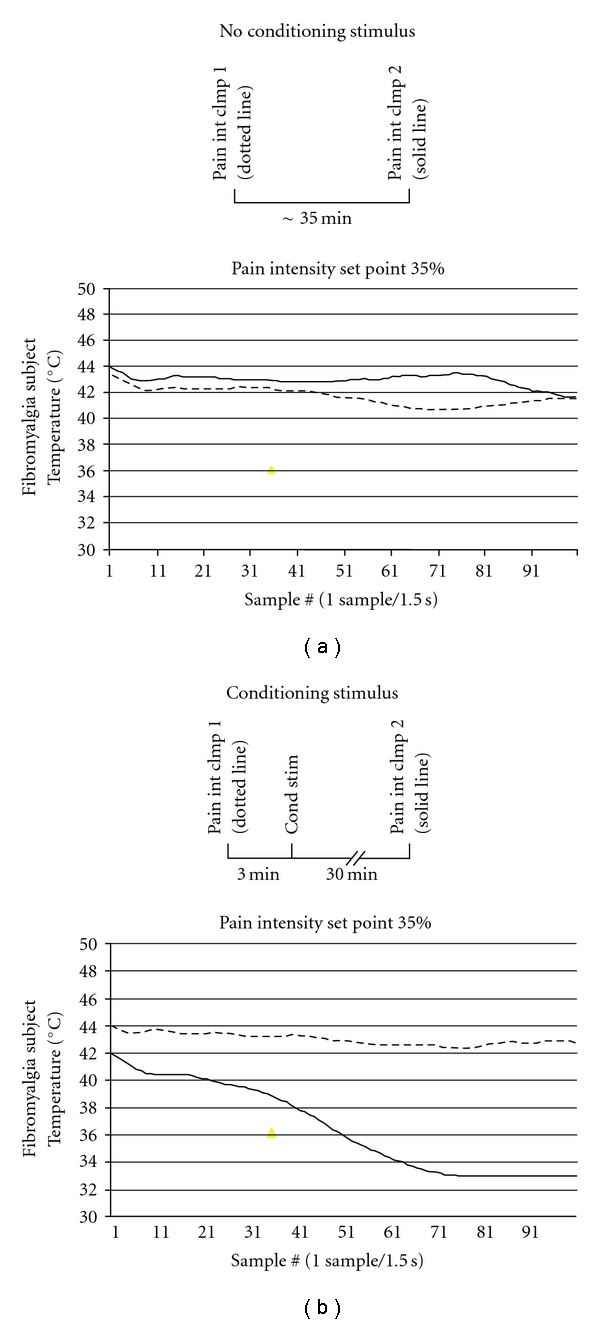
The FMS patient received 2 pain intensity clamping series (first series: dotted line; second series: solid line). The thermode remained in contact with the skin throughout the session, and the temperature was automatically adjusted to maintain an eVAS rating of 35%. The experiment was conducted with (b) and without (a) an intervening series of more painful stimuli (conditioning stimulus). The intervening conditioning stimuli sensitized the patient, as evidenced by a progressively decreasing temperature during the second 35% clamping test.

In all tests described thus far, the thermal stimulus was accompanied by mechanical contact of the thermode with the skin, so that discrimination between mechanical and thermal allodynia could not be made. Therefore, pain sensitivity was probed with a purely thermal stimulus. First, a two-minute period of continuous thermal stimulation (dotted lines in Figure 7) was administered with the thermode temperature automatically adjusted up and down in 1.5 sec intervals to maintain eVAS ratings near a 35% setpoint. The size of each adjustment step was a function of the difference of the pain intensity rating from the setpoint (proportional control) and the direction and magnitude of the trend (derivative control). This method of intensity adjustment permits long duration stimulation while maintaining sensation intensity at a desired and tolerable level as sensitization or adaptation progresses. The test was repeated 35 minutes later (solid line in Figure 7) with or without an intervening series of more painful stimuli. During the intervening series of thermal pulses (PD 3 sec, ISI 30 sec), the temperature increased in 0.5°C steps from 43 to 49°C. 

### 7.2. Results

During the repeat tests of 35% clamping (solid lines in Figure 7), the FMS subject exhibited progressive sensitization (a decrease in temperature to maintain ratings of 35%) only after the intervening series of painful stimuli Figure 7(b). Toward the end of the test after the intervening series, no more than 33°C was required to maintain 35% pain intensity. Thus, mild thermal pain (the first series of 35% clamping) did not affect ratings during a second series of 35% clamping Figure 7(a), but strong intervening pain substantially sensitized the patient, as revealed during a second series of 35% clamping. There was no contribution of mechanical stimulation to this effect.

## 8. Discussion

An individual with a long history of headache and TMJ pain presented with the diagnostic features of FMS. She is remarkably free of mental health comorbidities (e.g., depression), and her FMS pain has not been alleviated by a variety of medications, including antidepressants. An etiological factor for establishment and maintenance of her generalized FMS pain and hypersensitivity may be chronic stress associated with focal headache and facial pain for much of her life. Her clinical profile included autonomic signs suggestive of chronic stress. Psychophysical studies showed that exposure to nociceptive stimulation, which triggers an acute stress reaction [14, 15], switches the pain processing of this FMS patient into a different mode of operation. Extreme temporal summation of cutaneous thermal pain was observed after receipt of a stimulus that elicits a moderate to high intensity of pain.

 The psychophysical tests reported here demonstrate that the allodynia of this patient cannot be characterized simply as a generalized exaggeration of normally encoded pain. Abnormally high ratings of sensation intensity were dictated by the intensity and timing of present and preceding stimulations when the patient was blind to changes in stimulus parameters within and between tests. Moderate to high levels of pain produced a dramatic and long-lasting sensitization for subsequent thermal stimulation. These relationships between stimulus parameters and pain intensity reflect abnormal temporal and intensive processing within pain pathways rather than (or in addition to) a heightened impact of nociceptive input to higher cortical centers for affective interpretation. Investigation of mechanisms of chronic pain can benefit from a thorough psychophysical examination of patients with pronounced symptoms.
